# Debridement, antibiotics, and implant retention combined with direct intra-articular antibiotic infusion in patients with acute hematogenous periprosthetic joint infection of the knee

**DOI:** 10.1186/s12891-021-04451-x

**Published:** 2021-06-18

**Authors:** Pruk Chaiyakit, Surapoj Meknavin, Natthapong Hongku, Ittiwat Onklin

**Affiliations:** Department of Orthopedics, Faculty of Medicine, Vajira Hospital, Navamindradhiraj University, Bangkok, Thailand

**Keywords:** Acute hematogenous, Periprosthetic joint infection, Intraarticular antibiotic infusion, DAIR, Total knee arthroplasty

## Abstract

**Background:**

Debridement, antibiotics, and implant retention (DAIR) is the recommended treatment for acute hematogenous periprosthetic joint infection (PJI) after total knee arthroplasty (TKA). However, DAIR is associated with a high percentage of unsuccessful outcomes. Since 2007, direct intra-articular antibiotic infusion, which can provide a high concentration of intra-articular antibiotic, has been used in combination with DAIR to improve treatment outcomes among patients in our institution. This study aimed to assess the outcomes of DAIR combined with direct intra-articular antibiotic infusion in patients who presented with acute hematogenous PJI after TKA.

**Methods:**

We reviewed the data of all patients diagnosed with acute hematogenous PJI after primary TKA (from 2008 to 2015) who received DAIR combined with direct intra-articular antibiotic infusion.

**Results:**

In total, 15 knees in 12 patients were semi-urgently treated with this method. The mean follow-up time was 93.3 (minimum: 56) months, and the longest follow-up time was 11 years. Two patients (*n* = 3 knees) had a well-functioning, non-infected prosthesis 6 and 10 years after the procedure. Two patients (*n* = 2 knees) had re-infection 2 and 5 years after surgery, and they required two-stage revision. None of the patients were lost to follow-up. Finally, 13 (86.6%) of 15 infected knees were successfully treated with this method.

**Conclusions:**

DAIR combined with direct intra-articular antibiotic infusion is an effective treatment for acute hematogenous PJI after TKA.

## Introduction

Prosthetic joint infection (PJI) is a serious complication that occurs after primary total knee arthroplasty (TKA), with incidence rates ranging from 0.5 to 2%. Moreover, it is a major cause of revision surgery after TKA, with an incidence rate of 25% in all cases [1, 2]. According to the Tsukayama classification [3], PJI was divided into four types, which are as follows: from intra-operative culture positive to chronic infection with recommended treatment option. Debridement, antibiotics, and implant retention (DAIR) combined with a 6-week intravenous antibiotic treatment is the recommended treatment for acute hematogenous infection in the previous well-functioning knee prosthesis [3]. Even with this treatment, the results were still inconsistent, with a successful implant retention rate ranging from 32.6 to 70% [4–6]. Although the results varied, some reports showed that early intervention (within 7 days) had better outcomes.

To improve the outcomes of PJI, several studies have used direct intra-articular antibiotic infusion to increase the concentration of intra-articular antibiotics [7–10]. A previous study used a subcutaneous implantable pump combined with open arthrotomy debridement [11], which resulted in 88% retention of prosthesis. Moreover, Whiteside et al. [8]. showed that the success rate of one-stage revision combined with 6-week intra-articular antibiotic infusion for the management of chronic infection after TKA, even methicillin-resistant *Staphylococcus aureus* infection, was 94%. Hence, a high intra-articular antibiotic concentration is important for the treatment of PJI. Moreover, the intra-articular antibiotic concentration was 10,000 times greater than the minimal inhibitory concentration and 750 times higher than that of the intravenous route, with an intra-articular half-life of 3.22 h. The trough levels of treatment once a day remained above the minimal inhibitory concentration in the joint fluid even 24 h after injection [8, 12].

In 2007, we developed a treatment protocol for our institution to improve the outcomes of PJI treatment particularly in cases of acute infection after primary TKA. To decrease bacterial load and the risk of mature biofilm formation caused by delayed treatment, semi-urgent surgeries are performed immediately after the patient’s condition improved. Moreover, high-dose intra-articular antibiotic infusion via an intra-articular catheter was adopted, and it was used in combination with standard DAIR. Therefore, the current study aimed to assess the treatment outcomes of high-dose intra-articular antibiotic infusion and DAIR.

## Methods

### Study design

This study was approved by the institution review board (COA 045/57). All procedures were performed in accordance with the relevant guidelines. Then, we retrospectively reviewed data collected from the medical records from July 2008 to July 2015. Our inclusion criteria included all patients with primary TKA who had acute hematogenous infection according to the Tsukayama classification [3] and who received DAIR combined with direct intra-articular antibiotic infusion. Meanwhile, patients diagnosed with chronic PJI at the time of treatment and those with acute PJI after revision TKA were excluded.

### Diagnosis of PJI

Other than the clinical symptoms and signs of knee infection, PJI was mainly diagnosed using the laboratory investigation criteria, which were as follows: elevated serum erythrocyte sedimentation rate (ESR; critical level: 30 mm/h) and serum C-reactive protein (CRP) levels (> 10 mg/L); synovial fluid of the knee was analyzed in all cases; white blood cell (WBC) count at > 3000 cell/mm^3^ and percentage of polymorphonuclear cell (PMN) (> 80% of neutrophil); and positive joint fluid culture result. A diagnosis of PJI was made only if 3 of 4 criteria were met. Plain radiograph images were meticulously reviewed to identify new osteolysis around the implant, which could be a sign of chronic PJI. If suspected, standard two-stage revision will be performed, and the patient will not be included in this study. According to the institution’s protocol, most patients with PJI were diagnosed within 12 h after admission to the hospital. Subsequently, DAIR and insertion of an intra-articular catheter could be performed within the next 12 h.

### Surgical treatment

**Fig. 1:**
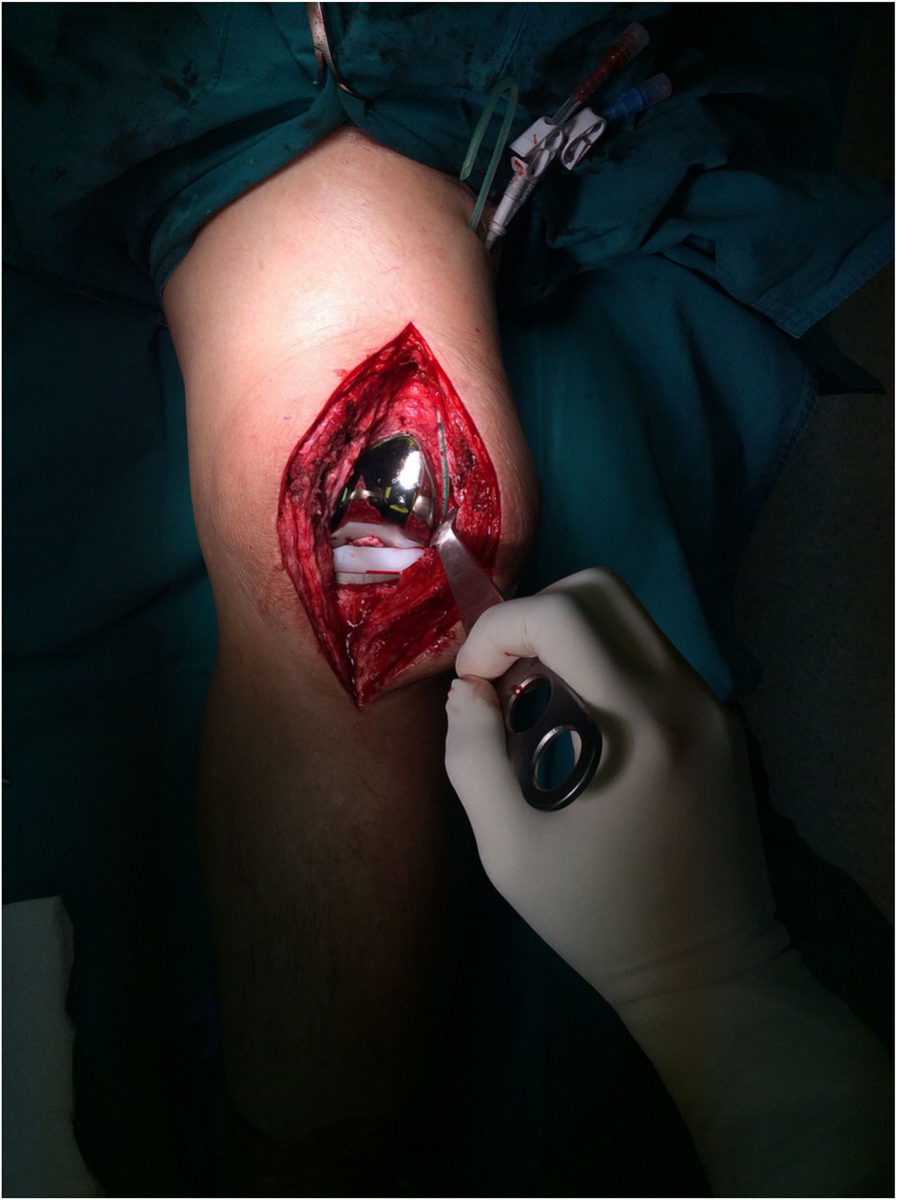
Intraarticular double-lumen catheter was placed into left knee joint

**Fig. 2:**
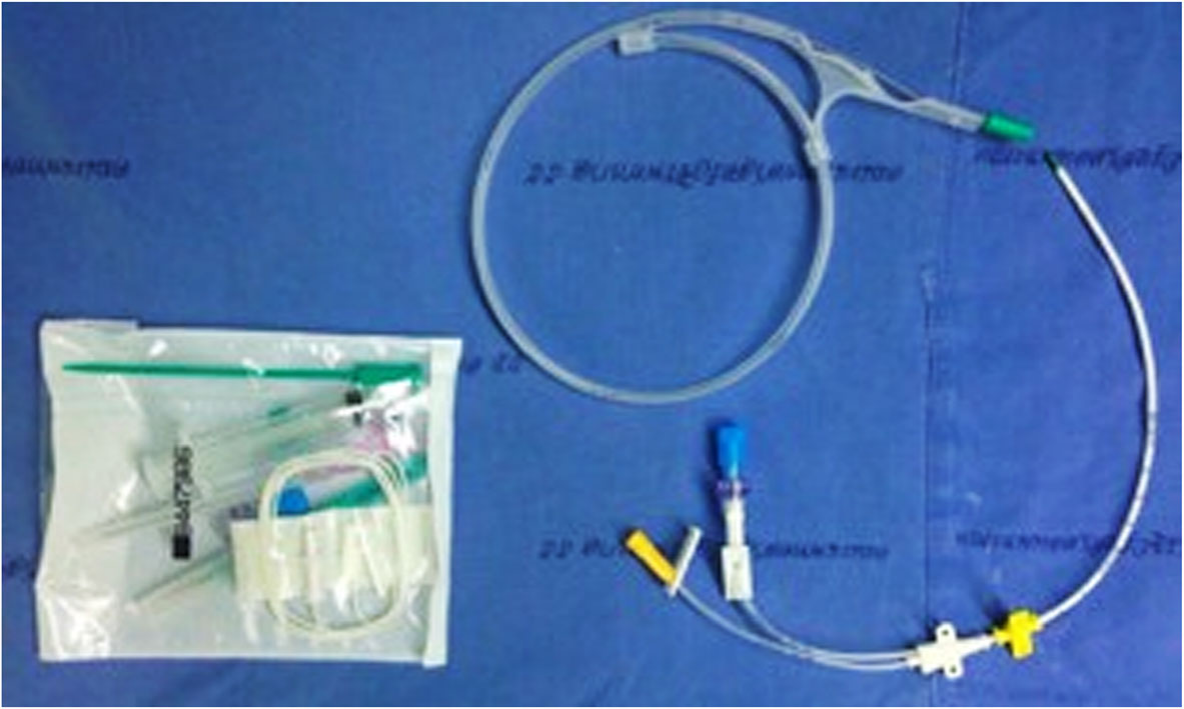
This figure illustrates double-lumen catheter which used in our study

The surgery started with standard open arthrotomy debridement and removal of polyethylene tibial insert. Debridement and initial irrigation with sterile saline solution (≥5 l) were cautiously performed; then, implantation with a new polyethylene liner was conducted. A catheter was placed into the knee joint, and the wound was closed normally (Fig. 1). We chose the double-lumen catheter (Fig. 2), which is normally used as an intra-articular catheter for central venous catheterization because of it is readily available in our hospital.

The first dose of intra-articular antibiotic was started in the operating room after wound closure based on previous culture or synovial fluid gram staining findings. The suction drain was used in all cases, and it was clamped for 4 h after surgery before removal within 48 h according to the standard protocol. After the removal of suction drain, antibiotic was infused daily via the catheter. Vancomycin (Siam Bheasach Ltd., Bangkok, Thailand) 100 mg diluted with 10 mL of sterile water was injected on the first day; then, the dose was increased to 100 mg daily, with a maximum at 500 mg. The local effect of vancomycin was observed until the patient was discharged. The duration of intra-articular infusion was 3 weeks. The function of the intra-articular catheter was checked daily before antibiotic administration. If there were any leakage or if obstruction is suspected, the catheter will be removed. Intravenous antibiotic was administered for 6 weeks. The type of antibiotic is based on culture sensitivity results or the presence of bacteria in gram staining. However, the duration of intravenous antibiotic treatment was reduced to 3 weeks and 1 week in 2011 and 2014, respectively. From 2014 onwards, all patients with positive culture results were discharged early, and they were instructed to go to the outpatient department daily for the infusion of intra-articular antibiotics for 3 weeks. Oral antibiotics were administered at a duration of 12 weeks according to current guideline [13]. The choice of oral antibiotics is based on the culture results. If the culture result finding was negative, first-generation cephalosporin was used.

After 3 weeks, the double-lumen catheter was removed. All patients were assessed for clinical signs of infection, and blood samples were collected for the evaluation of serum ESR and serum CRP levels weekly for 4 weeks, monthly for 3 months, and then every 3 months for 1 year. After 1 year, all patients were scheduled for annual follow-up or telephone consult in case they find it difficult to visit. The plain radiograph image of the knee was evaluated at 3 months and 1 year after DAIR. If there were clinical signs indicative of recurrent PJI, all laboratory results were evaluated to identify any infections. In cases of re-infection, two-stage revision was performed. Demographic, clinical, and laboratory data were collected. Treatment failure was defined using the following criteria: (1) clinical signs such as a non-healed wound with or without sinus tract, wound drainage, and persistent pain; (2) need for any surgical interventions for infection; (3) PJI-related mortality; and (4) need for long-term discontinuation of antibiotic treatment (> 6 months).

## Results

From July 2008 to July 2015, 15 knees in 12 patients were diagnosed with acute hematogenous infection after TKA. Three (25%) patients presented with bilateral knee infection. Moreover, there were 3 men and 9 women, with a mean age of 65.9 (range: 48.0–82.0) years, and the mean body mass index was 27.7 (22.9–42.0) kg/m^2^. Six (50.0%) patients had diabetes mellitus. None of patient receive antibiotic-loaded cement in primary TKA. The baseline characteristics and ASA physical status of all patients are summarized in Table 1.

**Table 1:** Demographic and Clinical data (12 Patients, 15 Knees)

Age (years)	65.9 (48–82)
Gender	
Male	3 (25%)
Female	9 (75%)
BMI (kg./m^2^)	27.7 (22.9–42.0)
ASA Classification	
I	3 (25%)
II	8 (67%)
III	1 (8%)
Comorbidities	
Diabetes	6 (50%)
Hypertension	10 (83%)
Chronic renal failure	1 (8%)
Rheumatoid arthritis	1 (8%)
Coronary artery disease	1 (8%)
Duration of symptom (hours)	107.2 (24–240)
Preoperative laboratory data	
Serum ESR (mm/hour)	92.7 (60–155)
Serum CRP (mg/L)	173.4 (14–381)
Synovial WBC (Cell/mm^3^)	52,736 (3400 - 140,000)
Synovial PMN (%)	96.1 (82–100)
Culture	
No growth	10 (66.7%)
Group A streptococcus	2 (13.3%)
Group B streptococcus	1 (6.7%)
*Staphylococcus aureus* (MRSA)	2 (13.3%)
Duration of Intraarticular injection (days)	17.5 (7–24)
Time to normal serum CRP (days)	71.4 (23–312)
Length of stay (days)	36.7 (4–66)
Follow up time (months)	93.3 (56–133)

*ASA* American Society of Anaesthesiologists, *BMI* body mass index, *MRSA* methicillin-resistant Staphylococcus aureus, *CRP* C-reactive protein, *ESR* erythrocyte sedimentation rate, *WBC* white blood cell, *PMN* polymorphonuclear neutrophils

The mean interval from the index TKA was 29.9 (range: 2–70) months. Only one patient underwent primary index TKA within 3 months. The patient had PJI, as evidenced by a high serum ESR (67 mm/h) and serum CRP level (381 mg/L). The PMN percentage was 94%, and the synovial WBC count was 65,700 cell/mm^3^. In addition, the culture was positive for Streptococcus group A. The mean ESR level was 92.7 (60–155) mm/h, and the mean serum CRP level was 173.4 (14–381) mg/L. The mean synovial WBC count was 52,736 (3400–140,000) cell/mm^3^, and the mean PMN percentage was 96.1% (82–100%). In total, five knees including two with methicillin-resistant *Staphylococcus aureus*, two with Streptococcus group A, and one with Streptococcus group B infections had positive culture results. All patients diagnosed with PJI at the time of surgery met at least three criteria.

The mean time from symptom onset to surgery was 107.2 (range: 24.0–240.0) h. The serum CRP level significantly decreased and normalized as early as 23 days, with an average of 71.4 days after surgery. The average duration of IA injection was 17.5 (range: 7–24) days. All patients were successfully treated based on this protocol, and they did not require further surgery. The average length of hospital stay was 36.7 (range: 4–66), and the mean follow-up time was 93.3 (range: 56–133) months. All clinical data are shown in Tables 2 and 3.

**Table 2:** Preoperative clinical data in 15 cases

	Age (yrs)	Sex	Site	BMI (kg/m^**2**^)	Comobidity	Preoperative serum CRP (mg/L)	Preoperative serum ESR (mm/h)	Preoperative Synovial WBC / %PMN
**Case 1**	72	Female	Right	25.8	CKD DM HT	82	106	21,700 / 96
**Case 2**	82	Female	Left	25.6	DM HT	14	73	54,000 / 97
**Case 3**	70	Male	Right	26.1	HT	381	67	27,000 / 98
**Case 4**	70	Male	Left	26.1	HT	381	67	65,700 / 94
**Case 5**	56	Male	Right	31.9	DM HT DLP	205	67	140,000 / 99
**Case 6**	58	Female	Left	42	HT	38	110	3400 / 82
**Case 7**	59	Male	Right	27.8	HT	299	123	40,500 / 97
**Case 8**	59	Male	Left	27.8	HT	299	123	76,000 / 97
**Case 9**	73	Female	Left	26	DM HT DLP	251	105	104,000 / 100
**Case 10**	71	Female	Left	23.8	DM HT	113	76	51,600 / 96
**Case 11**	48	Female	Left	25.6	HT	80	100	70,000 / 98
**Case 12**	72	Female	Right	36.9	RA	233	63	80,000 / 90
**Case 13**	72	Female	Left	22.9	RA	134	95	24,500 / 99
**Case 14**	69	Female	Right	24.2	DM HT CAD	56	60	17,600 / 99
**Case 15**	58	Female	Right	23.1	AIHA	35	155	15,040 / 99

*BMI* body mass index, *DM* diabetes mellitus, *HT* hypertension, *DLP* dyslipidemia, *CKD* chronic kidney disease, *RA* rheumatoid arthritis, *CAD* coronary arterial disease, *AIHA* autoimmune hemolytic anemia, *CRP* C-reactive protein, *ESR* erythrocyte sedimentation rate, *WBC* white blood cell, *PMN* polymorphonuclear neutrophils

**Table 3:** Details of clinical data in 15 cases

	Duration of intraarticular antibiotics	Culture	Intravenous antibiotics	Intravenous antibiotic duration (Days)	Oral antibiotics	Oral antibiotic duration (Days)	Time to normal serum CRP (Days)	Follow up time (Months)
**Case 1**	21	Negative	Fosfomycin	30	Amoxicillin and Clavulanate	30	37	99
**Case 2**	21	Negative	Fosfomycin	34	Ciprofloxacin	90	312	125
**Case 3**	20	Streptococcus gr. A	Penicillin G and Clindamycin	45	Cefdinir	120	111	133
**Case 4**	20	Streptococcus gr. A	Penicillin G and Clindamycin	45	Cefdinir	120	111	133
**Case 5**	13	Streptococcus gr. B	Fosfomycin	45	Amoxicillin and Clavulanate	90	113	110
**Case 6**	20	Negative	Cefazolin	5	Amoxicillin and Clavulanate	180	29	70
**Case 7**	14	Negative	Cefazolin	45	Cephalexin	30	35	117
**Case 8**	15	Negative	Cefazolin	45	Cephalexin	30	35	117
**Case 9**	24	Negative	Cefazolin	38	Amoxicillin and Clavulanate	90	26	65
**Case 10**	7	Negative	Ciprofloxacin	24	Ciprofloxacin	30	35	62
**Case 11**	14	Negative	Cefazolin	14	Amoxicillin and Clavulanate	60	34	65
**Case 12**	12	MRSA	Vancomycin	45	Cephalexin	60	28	72
**Case 13**	19	MRSA	Vancomycin	45	Fucidin	60	107	72
**Case 14**	21	Negative	Cefazolin	14	Amoxicillin and Clavulanate	60	35	103
**Case 15**	21	Negative	Cefazolin	14	Amoxicillin and Clavulanate	60	23	56

*MRSA* methicillin-resistant Staphylococcus aureus

After catheter removal, all patients had minimal drainage in the catheter site for a few days, and none had prolonged drainage (> 5 days). There was no complication in the catheter site and catheter-related events. All patients regained their previous activity level. Thereafter, oral antibiotic was administered for 12 weeks.

None of the patients were lost to follow-up in this study. Two (*n* = 3 knees) patients had an intact prosthesis after 6 and 10 years. Two patients with negative culture results (*n* = 2 knees) developed symptoms of loosening and underwent two-stage revision. A patient with colon cancer who is receiving chemotherapy first presented with re-infection, which developed to fungal PJI during the course of chemotherapy. However, the patient was symptom-free for 2 years after DAIR. Moreover, a patient with diabetes developed septic loosening after 5 years of DAIR. Finally, 13 (86.66%) of 15 infected knees were successfully treated with this technique.

## Discussion

PJI has been an increasing concern in arthroplasty practice nowadays. Since the average age of the population is increasing, a higher proportion of elderly patients will undergo TKA. With the presence of concomitant diseases such as diabetes, chronic kidney disease, and cancer, treatment could decrease the immune response of patients who undergo TKA, making them more susceptible to infection [14, 15]. The burden of PJI treatment did not only affect the patient’s health but also patient’s and stakeholder economics as well. Recently, Haddad et al. performed a cost analysis of DAIR and two-staged revision. Results showed that the cost saving from treatment with DAIR within 1 year could reach up to approximately 55,0000,000 USD. Its cost was about one-third of that of two-staged revision [16–18]. However, the medical fee is significantly higher in our institution.

Generally, PJI treatment is chosen based on duration of symptoms, which indicated biofilm formation. In case of acute PJI, such as acute postoperative and acute hematogenous, the duration of infection is < 4 weeks. This will then lead to the formation of immature biofilms. In this group, DAIR was the recommended treatment. However, chronic PJI is correlated with mature biofilm formation, which requires implant removal for a thorough debridement of a mature biofilm [19]. The early diagnosis of acute PJI is important because delayed diagnosis could result in a higher failure rate, as shown in previous reports. Therefore, early intervention had better outcomes [20, 21]. This result could be explained by bacterial growth and biofilm formation. Delayed diagnosis and treatment will eventually increase bacterial load over time and the risk of biofilm formation and will inevitably lead to the development of chronic PJI.

DAIR was the treatment of choice for acute PJI because it was a strategic and economic approach. Although two-stage revision arthroplasty had a higher success rate, the overall treatment cost is significantly higher, and re-infection still occurs after the procedure. With consideration of economic burden, recent studies have found that DAIR is more cost-effective than two-stage revision arthroplasty [16–18]. However, the former has a significantly lower efficacy than the later. Several factors could lead to DAIR treatment failure. These include misdiagnosis of acute PJI and miss management with DAIR if it was indeed a case of chronic infection. Further, delayed diagnosis and treatment lead to the development of more mature biofilm, which result in poorer outcomes [21, 22]. Apart from diagnostic challenges, changing all exchangeable parts can provide better outcomes. However, debridement with retention of the prosthesis in situ was technically demanding. Thus, it is associated with a higher risk of inadequate debridement compared with two-stage debridement [23, 24].

The efficacy of infection eradication can locally affect failure to maintain an adequate level of antibiotic locally. Some antibiotics, including beta-lactams, have a lower intra-osseous concentration than others. The sub-therapeutic level of beta-lactams could cause biofilm formation in *S. aureus* organisms [25]. A higher-dose intra-articular antibiotic infusion via an intra-articular catheter could play an important role by maintaining the therapeutic level of intra-articular antibiotic even with treatment once a day [8, 12]. Therefore, strategies including early diagnosis and intervention and high-dose intra-articular antibiotic infusion via an intra-articular catheter can improve treatment outcomes. With combined DAIR and intra-articular antibiotic infusion via an intra-articular catheter, we could successfully retain 86.66% of infected prostheses.

Retaining an intra-articular catheter might be associated with catheter-related events, which lead to occult infection and recurrent periprosthetic joint infection. There was no catheter-related event in our study, and it is rarely reported in previous studies. However, there were two cases of treatment failure, which were not correlated to the use of a catheter.

The current study had several limitations. First, we obtained a relatively high proportion of negative culture findings, which reflected the disadvantages of such technique during that period and caused empirical antibiotic treatment about two-third of the cases. However, in our study, the diagnosis of PJI was based on clinical and laboratory studies, which were in accordance with the MSIS criteria 2014 and 2018. All patients diagnosed with PJI met at least three minor criteria. Second, the number of cases was quite small due to the nature of the disease. However, none of the patients were lost to follow-up.

## Conclusion

DAIR combined with 3-week direct intra-articular antibiotic infusion via a double-lumen catheter is an effective treatment for acute hematogenous infections after TKA. Early debridement and maintenance of high-dose intra-articular antibiotic infusion could be key factors in the eradication of causative organisms, which leads to a successful treatment of PJI type III.

## Data Availability

The datasets used and/or analysed during the current study available from the corresponding author on reasonable request.

## Abbreviations

DAIR: Debridement, antibiotics, and implant retention
PJI: Periprosthetic joint infection
TKA: Total knee arthroplasty
ESR: Erythrocyte sedimentation rate
CRP: C-reactive protein
WBC: White blood cell count
PMN: Polymorphonuclear cell
